# Reasons For Physicians Not Adopting Clinical Decision Support Systems: Critical Analysis

**DOI:** 10.2196/medinform.8912

**Published:** 2018-04-18

**Authors:** Saif Khairat, David Marc, William Crosby, Ali Al Sanousi

**Affiliations:** ^1^ Carolina Health Informatics Program University of North Carolina at Chapel Hill Chapel Hill, NC United States; ^2^ School of Nursing University of North Carolina at Chapel Hill Chapel Hill, NC United States; ^3^ Health Informatics Graduate Program College of Saint Scholastica Duluth, MN United States; ^4^ Hamad Medical Coperation Doha Qatar

**Keywords:** decision support systems, clinical, decision making, computer-assisted, attitude to computers

## Abstract

**Background:**

Clinical decision support systems (CDSSs) are an integral component of today’s health information technologies. They assist with interpretation, diagnosis, and treatment. A CDSS can be embedded throughout the patient safety continuum providing reminders, recommendations, and alerts to health care providers. Although CDSSs have been shown to reduce medical errors and improve patient outcomes, they have fallen short of their full potential. User acceptance has been identified as one of the potential reasons for this shortfall.

**Objective:**

The purpose of this paper was to conduct a critical review and task analysis of CDSS research and to develop a new framework for CDSS design in order to achieve user acceptance.

**Methods:**

A critical review of CDSS papers was conducted with a focus on user acceptance. To gain a greater understanding of the problems associated with CDSS acceptance, we conducted a task analysis to identify and describe the goals, user input, system output, knowledge requirements, and constraints from two different perspectives: the machine (ie, the CDSS engine) and the user (ie, the physician).

**Results:**

Favorability of CDSSs was based on user acceptance of clinical guidelines, reminders, alerts, and diagnostic suggestions. We propose two models: (1) the user acceptance and system adaptation design model, which includes optimizing CDSS design based on user needs/expectations, and (2) the input-process-output-engagemodel, which reveals to users the processes that govern CDSS outputs.

**Conclusions:**

This research demonstrates that the incorporation of the proposed models will improve user acceptance to support the beneficial effects of CDSSs adoption. Ultimately, if a user does not accept technology, this not only poses a threat to the use of the technology but can also pose a threat to the health and well-being of patients.

## Introduction

The Agency for Healthcare Research and Quality [1] promotes a systems approach that aims “to catch human errors before they occur or block them from causing harm.” Clinical decision support systems (CDSSs) are at the forefront of this aim. A CDSS provides alerts, reminders, prescribing recommendations, therapeutic guidelines, image interpretation, and diagnostic assistance. Although studies have shown that CDSSs reduce medical errors and improve outcomes, they also demonstrate that CDSSs fall short of their full potential [2-9]. Research has attempted to narrow in on the cause of this shortfall. Coiera [10] identified provider’s lack of willingness and ability to use the technological system as one of the primary reasons.

Wendt et al [11] discussed several factors that may be related to the acceptance of CDSSs, including the relevance of the information provided by the system, perceived validity of the system, and the work and time expended on using the system. These factors are similar to those defined by Davis [9] in the technology acceptance model (TAM) and later refined by Venkatesh et al [12] in the unified theory of acceptance and use of technology (UTAUT). These models offer a potential explanation for how expectations of performance, effort, social influences, and facilitating conditions are determinants of user acceptance and technology usage [12]. Using the TAM, Van Schaik et al [13] evaluated a gastroenterology referral CDSS. The system assisted primary care providers by suggesting an appropriate subspecialty referral (medical vs surgical), prioritizing urgency, and offering real-time booking [13]. They found that physicians rated acceptance based on the potential merits of the system rather than their experience with the computer system [13]. This finding was concordant with Venkatesh et al’s [12] proposal of the UTAUT model, in which they demonstrated that performance expectancy is the strongest predictor of user acceptance of technology.

The theory behind user acceptance and its impact on the adoption of technology has been thoroughly described. The purpose of this paper was to conduct a review of the literature in order to evaluate our hypothesis that meaningful engagement of physicians in the design and development of CDSSs with transparent decision-making processes will result in higher acceptance rates.

## Methods

### Critical Review

**Figure 1:**
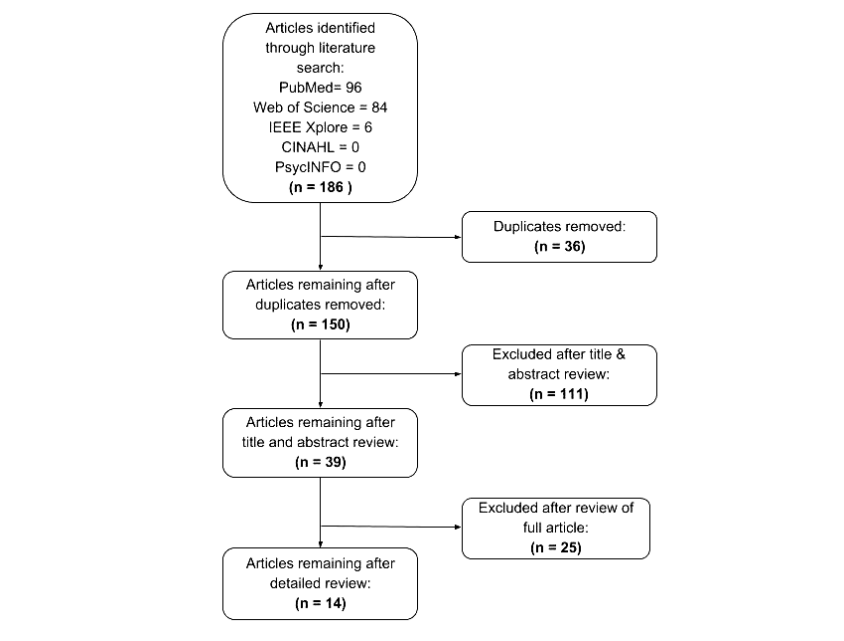
Preferred Reporting Items for Systematic Reviews and Meta-Analyses (PRISMA) Diagram.

A search of MEDLINE/PubMed, CINAHL, PsycInfo, IEEE Xplore, and Web of Science was conducted using the keywords “clinical decision support,” “decision support acceptance,” and “user acceptance.” No timeframe limits were included for any database, and the language filters were set to English studies only. In our initial search, we found 186 papers. After removal of duplicates, 150 studies remained. To be included in this review, the papers had to match the following inclusionary criteria: investigate human interaction with a CDSS and evaluate user acceptance using the TAM questionnaire, focus groups, or interviews. Papers were excluded if the focus was on decision support systems that did not include clinical care or if they did not empirically investigate user acceptance. Title and abstract review eliminated 111 studies. The remaining 39 studies underwent a full-text review, resulting in a final count of 14 studies that met inclusion criteria. The search results are summarized in Figure 1.

Study findings were categorized as either showing favorable or unfavorable responses to CDSSs. The favorable and unfavorable categorization was based on interpretations of focus groups and interviews conducted by the researchers of the reviewed papers. Additionally, the type of CDSS was noted for each of the reviewed papers. If a study used the TAM questionnaire, the results were summarized separately.

### Task Analysis

To gain a greater understanding of the problems associated with CDSSs, we conducted a task analysis. Using past research, the task analysis helped identify and describe the goals, user input, system output, knowledge requirements, and constraints from two different perspectives. We considered the perspective of the machine (ie, the CDSS engine). We also considered the perspective of the user (ie, the physician). The literature review and task analysis served as the basis for designing CDSS models that improved user acceptance.

## Results

### Critical Review

The results of 14 articles were evaluated. The 11 articles that qualitatively evaluated user acceptance of CDSSs can be found in Table 1 and the three articles that quantitatively evaluated user acceptance of CDSSs using TAM can be found in Table 2. Favorable and unfavorable responses for the aspects of clinical guidelines, reminders, and diagnostic CDSSs were recorded. Favorable responses were due to ease of system use, perceived time savings, and perceived usefulness of the systems in improving care delivery and overall patient health [14]. Users with higher computer skills were reported to have greater acceptance; however, the majority of users had an unfavorable acceptance response [15]. These unfavorable responses were often related to workflow interference, questionable validity of the systems, excessive disturbances caused by the systems, and lack of efficiency. More specifically, the workflow constraints were related to the CDSSs causing excessive alerts, increased time in computer handling, and decreased face-to-face time with patients [14,16,17].

Of the studies reviewed, three used the TAM questionnaire for assessing user acceptance of CDSSs (Table 2). The CDSSs in these studies included an evaluation of two different computerized clinical guideline systems and one that offered reminders and alerts for evidenced-based guidelines. The ranges on perceived usefulness all overlap, but on the CDSSs with the highest perceived usefulness it also has the highest perceived ease of use. Overall, the TAM questionnaire revealed moderate user acceptance on all scales. In terms of the relationship between user acceptance of CDSSs and patient safety, none of the reviewed papers evaluated this topic. However, Bergman and Fors [15] found that the use of the technology was relatively low when user acceptance was low.

**Table 1:** Summary of user acceptance related to clinical decision support systems (CDSSs) from previous studies (N=11).

Study	Favorable response to CDSS	Unfavorable response to CDSS	CDSS Description
Bergman & Fors (2005) [15]	Can save time and provide structure	Not suitable to workflow and there is the risk of becoming dependent	CDSS for medical diagnosis of psychiatric diseases
Curry & Reed (2011) [16]	Concept was supported	Interference with workflow and questionable validity	Prompts for adhering to diagnostic imaging guidelines
Gadd et al (1998) [18]	Easy to use, limits the need for data entry, accurate, and relevant	Benefits are lost because it takes so long to use	Internet-based system that interactively presents clinical practice guidelines at point of care
Johnson et al (2014) [19]	Longitudinal acceptance behavior, perceived ease of use, and perceived usefulness	Computer literacy, user satisfaction, and general optimism	Clinical reminders and alerts for patients with asthma, diabetes, hypertension, and hyperlipidemia
Rosenbloom et al (2004) [20]	Can improve efficiency and quality of care; enhances education	Senior physicians did not think it was necessary	CDSS for computerized order entry system
Rousseau et al (2003) [21]	Use of “active” CDSS can bridge the gap between own practice and best practice	Clinicians found it to be difficult to use and unhelpful clinically	CDSS for chronic disease in general practice
Shibl et al (2013) [22]	Performance expectancy, usefulness, and effort expectancy	Trust in CDSS and need for the system	No specified CDSS; responses based on past and present experiences with multiple CDSSs
Sousa et al (2015) [23]	Belief that the suggestions were good for the patient	Low confidence in the evidence	CDSS for nursing care plan
Terraz et al (2005) [24]	Ease of use and easy access to information	Information that is presented is already known	Guidelines for colonoscopies
Wallace et al (1995) [25]	Can improve patient outcomes	Alerts are ignored because there is not enough time to dedicate to forming an appropriate response	CDSS to standardize administration of supplemental oxygen
Zheng et al (2005) [17]	Improves performance leading to better care, easy to use, and efficient	Iterative advisories, lack of relevance, a lot of data entry, and disruptive	Clinical reminders for chronic diseases and preventive care

**Table 2:** Results of the technology acceptance model (TAM) questionnaire from prior studies evaluating user acceptance of CDSSs.

Study		Buenestado et al (2013) [26]	Heselmans et al (2012) [27]	Peleg et al (2009) [28]
CDSS description		Computerized clinical guidelines and protocols for asthma in children	Reminders and alerts for evidenced-based guidelines	Guideline-based decision support system for diabetic patient foot problems
Participant description		8 pediatricians	39 Dutch-speaking family physicians	8 family physicians
**Likert scale score^a^, mean (SD)**		Seven-point scale	Seven-point scale	Five-point scale
	Perceived usefulness	5.80 (1.24)	4.00 (1.37)	4.00 (0.71)
	Perceived ease of use	6.17 (0.92)	5.02 (1.41)	4.40 (0.59)
	Attitude toward using	6.21 (0.59)	4.84 (0.97)	N/A
	Behavioral intention to use	5.71 (1.24)	5.91 (1.33)	4.88 (0.23)

^a^The scores are based on a Likert scale (1=totally disagree; 5 or 7=totally agree).

### Task Analysis

Task analysis is conducted to stay updated with the changing professional practice (ie, health information technology) [29]. Task analysis applied to representative populations strengthen health systems by systematically evaluating the skills, knowledge, and behavior of clinicians that impact clinical practice [30]. The use of CDSS in health care has introduced new dynamics to practice and requires task analysis to understand the perception of users to this new technology. For that reason, conducting a task analysis will improve adoption levels. A task analysis includes goals, input, process, and output. The next sections discuss the purpose of each task analysis stage.

#### Goals

The goal of a CDSS is to supplement the physician as the sole information processor in clinical decision making and thereby aid in the reduction of medical errors. Yet, there is still much room for improvement. In part, this shortcoming may be due to the lack of physician acceptance of the CDSSs in supplementing their decision making. To get a better understanding of the challenges in creating clinical decision processes, we first consider what information goes into this process.

#### Input

A CDSS is based on an input-process-output (IPO) model. The inputs for the CDSS process include patient-specific information such as diagnoses, medications, symptoms, laboratory data, demographics, and other clinically relevant information. The inputs for knowledge-based CDSSs are often determined by clinical guidelines, whereas non-knowledge-based CDSSs use the most relevant information assessed by algorithm performance.

#### Process

The CDSS process takes two different forms: knowledge based and non-knowledge based [31]. Knowledge-based systems are governed by a set of rules. Non-knowledge-based systems, on the other hand, use a computer as the central processing unit to learn from historical information. As a result, these systems typically utilize machine learning algorithms.

When CDSSs offer clinical suggestions, the support, evidence, clinical guideline, or algorithm for those suggestions is not provided. The inputs for knowledge-based CDSSs are often determined by clinical guidelines, whereas non-knowledge-based CDSSs use the most relevant information assessed by algorithm performance. In both cases, the physician is not aware of the inputs or processes the CDSS utilizes. Thus, the CDSS is a black box to the physician.

Physicians make clinical decisions based on the same patient information in addition to social structures (acceptable behavior as determined by peer groups), institutions (the requirement to act according to mandated practices), and individual morality in decision making. One can conjecture that difficulties arise when automating such a complex network of inputs that could never by fully encapsulated or realized by a machine.

#### Output

The output from CDSSs and physicians are a result of the methods employed for processing the inputs. The output may be a diagnosis, procedure, prescription, etc. Ideally, in conditions where the computer and the physician are presented with the same information, the output from the CDSS should mirror the physician’s decision.

The level of control the CDSS has with regards to the output is inversely related to the level of control the user has over the output. A CDSS can be passive in situations where they only “highlight” information for the user, but do not request acknowledgment or action [32]. An example would be presenting abnormal laboratory values in a red font and normal laboratory values in a black font. Active CDSSs act independently and provide suggestions to guide the physician’s behavior [32]. An example would be a system that provides diagnostic assistance. The type of output then depends on the goal orientation of a task (eg, diagnoses, medication alerts, and clinical guidelines for preventive care).

### Knowledge

Physicians are more likely to accept a CDSS if the system matches their own decision-making processes. Forster [33] described how humans quickly act on information by using bounded and ecological rationality. Bounded rationality is based on the use of simple heuristics, allowing for fast, real-time decision making [34]. Ecological rationality is based on rational beliefs of things in a given environmental setting where conditions are fluid. Forster [33] argued that both bounded and ecological rationality need to be present in machine learning to mimic the human decision processes.

Incorporating these two approaches into CDSSs can be challenging. Even though the heuristics that mediate decision processes are simple; the complexity of the cognitive infrastructure underlying heuristic operations can be difficult to implement. Still, Forster [33] argued that machine learning algorithms can be improved by incorporating the principles of bounded and ecological rationality. To carry out this task, Forster [33] suggested that a decision-making machine should have (1) a set of ad hoc rules (or biases) to act on and (2) a set of ecologically viable environmental factors to consider.

Clark [35] extended this idea of mediating decision processes by bounded and ecological rationality through a concept he referred to as scaffolding. Clark [35] posited that human reasoning involves three aspects: (1) individual reasoning cast by some form of fast, pattern-completing style of computation (ie, bounded rationality); (2) substantial problem-solving work offloaded onto external structures and processes (eg, social and institutional structures); and (3) public language used as a means of coordinating social structures and mediating individual thought. Thus, decision making and cognition are largely dependent on the capacity to dissipate reasoning throughout the environment to reduce individual workload.

Holland and colleagues [36] added additional elements that can be useful to understand physician decision making. These elements provide a cognitive framework for problem solving, which includes two distinct schemas: pragmatic reasoning schema and problem schema. Pragmatic reasoning schemas are clusters of abstract inferential rules that characterize relations over general classes of object kinds, event relationships, and problem goals. Problem schemas are used by experts to solve routine problems, where an expert retrieves an appropriate problem schema and provides it with problem-specific parameters.

The system must also have two types of knowledge structures: mental models and condition-action rules. Holland and colleagues [36] assert that “mental models are transient, dynamic representations of particular, unique situations. They exist only implicitly, corresponding to the organized, multifaceted description of the current situation and the expectations that flow from it.” A condition-action rule can be thought of as an IF (condition)...THEN (action) statement. Together, these knowledge structures allow the mental schemas to operate in order to solve problems.

To successfully implement and use CDSSs, these mental models have to be identified. Hayek [37] stated that knowledge is not given to anyone in its entirety. This statement legitimizes why CDSSs are so important. In theory, CDSSs lessen the cognitive resources a physician needs to make decisions.

### Constraints

The major limitation of CDSSs is that scaffolding cannot be fully captured by computers. The environmental, clinical, and social constraints in which physicians practice are difficult to include as inputs into a CDSS. In addition, reproducing a physician’s tacit knowledge through mental models and condition-action rules is a formidable objective. Additionally, physicians must be able to support their decision and are skeptical of recommendations or claims that lack supporting evidence or transparency. The fact that CDSSs do not reveal how output decisions are made may be a driving force behind the lack of users’ acceptance.

## Discussion

### Means for Solving User Acceptance of Clinical Decision Support Systems

Studies have revealed that responses to CDSSs can be unfavorable when resulting improvements in patient outcomes are inconsistent [6]. Also, some studies have reported incidents of patient harm associated with CDSS implementation [38]. Despite these findings, limited research has formally evaluated the impact of user acceptance. Based on our comprehensive review of the literature, we have found both favorable and unfavorable user acceptance to CDSSs.

If a user finds a product frustrating or perceives that the purpose of the product is to limit autonomy, the user may not use the product or do so inappropriately [39]. Vashitz et al [40] explains the consequence of loss of autonomy as reactance. *Reactance* is an unpleasant motivational state whereby people react to situations to retain freedom and autonomy. Reactance may exist when physicians feel threatened by clinical reminders for fear that they are losing autonomy and freedom of choice in the presence of such systems. Physicians may have the perception that these systems are meant to replace or degrade their clinical duties. Vashitz et al [40] describe how unsolicited advice may lead to a reactance state if the advice contradicts a person’s original impression of choice options.

Based on the UTAUT, user expectations need to be taken into consideration for technology to be accepted [12]. Therefore, in the design of CDSSs, the human element cannot be ignored. Reminders and alerts should be presented in such a way that the user does not find them threatening or obtrusive. User needs and expectations of a CDSS should be evaluated early and throughout the development lifecycle. For instance, Gadd [18] observed enhanced usability and usefulness by implementing usability testing in the early phases of CDSS development. They evaluated an evolving prototype of the system and observed user interactions over a 3-month period. In a series of sessions, they focused on evaluating user interactions with different sets of system features such as screen layout, input/output, and links to educational materials. Finally, they considered the user feedback on system recommendations in the design process. Compelling suggestions for system enhancements made by users during the earlier sessions influenced system development of features that were evaluated in later sessions.

Peleg et al [28] discussed the development process of their CDSS, where clinically knowledgeable users worked alongside the developers to design and implement the CDSS. They also used a lifecycle model user-centered design and evaluation process for evaluating the users’ goals/expectations, workflow, environmental constraints, and tasks. Finally, they conducted usability testing (ie, heuristic evaluation of user-interface, keystroke-level modeling, and cognitive walkthroughs) prior to implementation.

Developers of CDSSs have attempted to bottle-up the decision-making capacity of physicians and place that knowledge into a computer. Current methods to achieve this feat employ rules and machine learning algorithms. However, the lack of user acceptance has impeded CDSS use. Research has shown that consideration of users’ needs and expectations in the design of the CDSS may help overcome this obstacle. We argue that this approach is only part of the solution.

We propose that CDSSs move away from the black-box process to a more transparent method within the IPO model. Simply put, tell the physician how the computer is making the decision. If the computer can become part of scaffolded knowledge, the physician may view the computer as an aid rather than a threat or hindrance. Research supports the idea that the rules governing alerts be specified to practitioners and the information be presented based on users’ needs and expectations [41].

### Proposal of Models to Gain User Acceptance

We propose two models to improve CDSSs development that may lead to increased utilization resulting in improved patient outcomes. First, is the user acceptance and system adaptation design (UASAD) model that aims to involve end users early in the design and throughout the development of CDSSs. Second, is replacing the current IPO model of CDSS development with the input-process-output-engage (IPOE) model that serves to “engage” the physician through CDSS process transparency.

The UASAD model demands early end-user involvement in CDSS development. User needs and expectations need to be fully realized prior to the development of a CDSS. Another consideration is to evaluate system preparedness to ensure that users can trust the security and privacy of the system. Prototypic designs should undergo an iterative design process following rigorous usability testing in a laboratory and natural setting (ie, pilot study) to ensure that the system works within the cognitive and environmental constraints with which the user functions.

**Figure 2:**
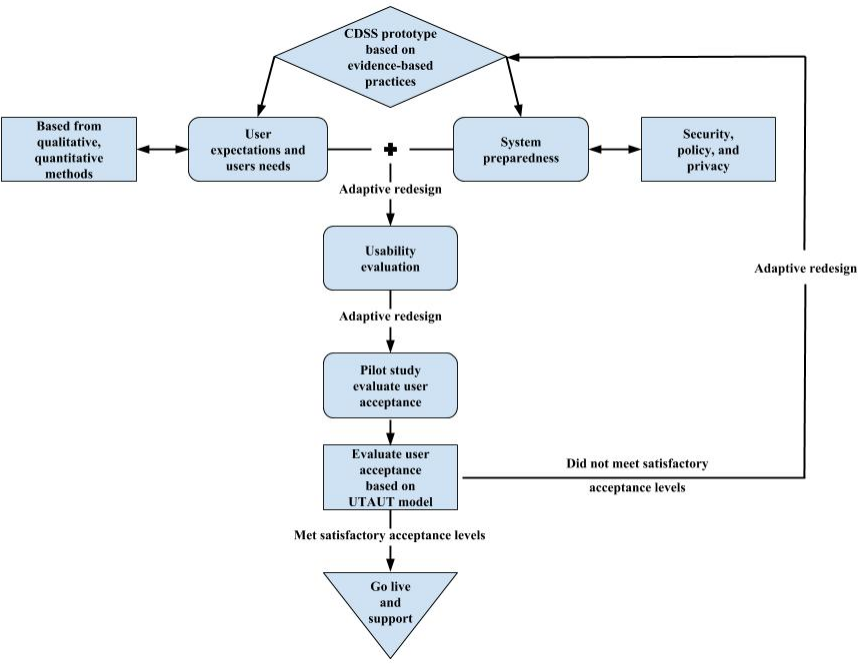
A user acceptance and system adaptation design (UASAD) model. CDSS: clinical decision support system; UTAUT: unified theory of acceptance and use of technology.

Finally, user acceptance should be evaluated to ensure that the system is used appropriately. If user acceptance is not achieved above a predefined threshold, the CDSS should be reevaluated from the point of view of user needs and expectations. It should also be subjected to adaptive redesign. This process should iterate until user acceptance exceeds a predefined threshold. To illustrate this process, we have developed a UASAD model (Figure 2). The purpose of the model is to include the user as the focal point of the design process of CDSS.

**Figure 3:**
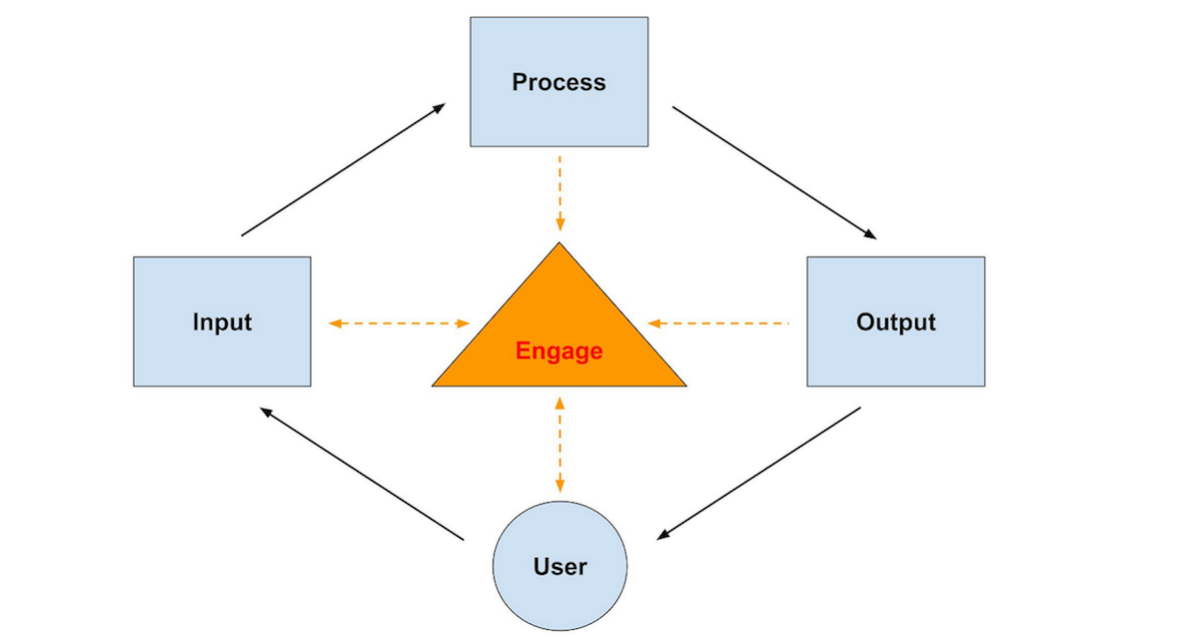
The input-process-output-engage (IPOE) model.

The IPOE model offers users a window into the black-box IPO process. Through “engage” physicians will see how the CDSS is making decisions. The IPOE window will be called “engage” because it will present users with the rules that the machine followed to generate the output (Figure 3). Therefore, the user can make informed decisions when determining to accept or deny outputs. “Engage” will display the input, process, and output that led to the CDSS’s decision. The physician will then be able to evaluate the relevancy, validity, supporting evidence, and strength of a recommendation. Therefore, this system becomes a component of the physician’s scaffolded knowledge and enables them to act more confidently in accepting the technology and its role in their decision-making processes.

A limitation of the IPOE model is that in order for the model to work successfully, the physician has to understand the process. Processes that utilize a machine learning algorithm, such as neural networks, do not provide rules. Therefore, it is challenging to make all processes transparent.

### Why Do We Make Bad Decisions?

Physicians’ tendencies to incorrectly process challenging decisions usually lead to bad clinical decisions. Most practicing physicians tend to make decisions out of their own medical experience, whereas others pursue medical consultations and filtering through the jargon of relevant research. The most effective physician, though, is the one who has the ability to utilize his clinical judgment coupled by the computerized decision support tools to leverage the power of CDSSs. Most clinicians exhibit bias when it comes to medical information that they know and, therefore, they typically focus on things that would agree with the specific clinical outcome that they want to see in their patients. Therefore, the context of using a CDSS is mandated by the efforts to decrease medical errors by utilizing existing knowledge and technology. These systems are a result of long-term scientific research to build efficient tools for physicians to supplement their clinical experience. Physicians should look at CDSSs as an added value to make the best decisions in their day-to-day practice and to better serve their patients. These systems seek to reduce medical errors by enabling the practicing physicians to make informed decisions that are both accurate and precise.

### Conclusion

Implementation of CDSSs has demonstrated increased efficiency, reduced medical errors, and improved outcomes, but they continue to fall short of their full potential [2-9]. We believe this key shortcoming may partly be due to the lack of physician acceptance. In the past, CDSS designs have not incorporated input from physicians and do not reveal their decision-making processes. Consequently, many physicians are hesitant to accept CDSSs leading to suboptimal implementation. Here we propose two models for designing CDSSs with the goal of improving efficacy and physician acceptance. One model, UASAD, focuses on including the physician in the design process by examining user needs and expectations and usability of prototypic designs. The other model, IPOE, extends the existing IPO framework by adding an “engage” stage that displays the CDSS process to the physician. This approach allows the physician to include the CDSS as a component of their decisions while maintaining professional autonomy. There is still considerable work to be done for validating these models, yet user acceptance appears to be pertinent for successful CDSS use. Ultimately, if a physician does not accept the technology, it not only poses a threat to the use of the technology but can also pose a threat to the health and well-being of patients.

## Abbreviations

CDSS: clinical decision support systems
IPO: input-process-output
IPOE: input-process-output-engage
TAM: technology acceptance model
UASAD: user acceptance and system adaptation design
UTAUT: unified theory of acceptance and use of technology
